# A cluster randomized controlled trial on a multifaceted implementation strategy to promote integrated palliative care in COPD: study protocol of the COMPASSION study

**DOI:** 10.1186/s12904-020-00657-3

**Published:** 2020-10-10

**Authors:** Johanna M. C. Broese, Rianne M. J. J. van der Kleij, Huib A. M. Kerstjens, Els M. L. Verschuur, Yvonne Engels, Niels H. Chavannes

**Affiliations:** 1 Public Health and Primary care, Leiden University Medical Centre, Post zone V0-P, Postbox 9600, 2300 RC Leiden, The Netherlands; 2 Lung Alliance Netherlands, Amersfoort, The Netherlands; 3 Department of Respiratory Medicine and Tuberculosis, and Groningen Research Institute for Asthma and COPD (GRIAC), University of Groningen and University Medical Centre Groningen, Groningen, The Netherlands; 4 Anaesthesiology, Pain and Palliative Medicine, Radboud University Medical Centre, Nijmegen, The Netherlands

**Keywords:** COPD, Exacerbation, Proactive palliative care, Advance care planning, Quality of life, Integrated care, Implementation study, Cluster randomized controlled trial

## Abstract

**Background:**

Despite the urgent need for palliative care for patients with advanced chronic obstructive pulmonary disease (COPD), it is not yet daily practice. Important factors influencing the provision of palliative care are adequate communication skills, knowing when to start palliative care and continuity of care. In the COMPASSION study, we address these factors by implementing an integrated palliative care approach for patients with COPD and their informal caregivers.

**Methods:**

An integrated palliative care intervention was developed based on existing guidelines, a literature review, and input from patient and professional organizations. To facilitate uptake of the intervention, a multifaceted implementation strategy was developed, comprising a toolbox, (communication) training, collaboration support, action planning and monitoring. Using a hybrid effectiveness-implementation type 2 design, this study aims to simultaneously evaluate the implementation process and effects on patient, informal caregiver and professional outcomes. In a cluster randomized controlled trial, eight hospital regions will be randomized to receive the integrated palliative care approach or to provide care as usual. Eligible patients are identified during hospitalization for an exacerbation using the Propal-COPD tool. The primary outcome is quality of life (FACIT-Pal) at 6 months. Secondary outcome measures include spiritual well-being, anxiety and depression, unplanned healthcare use, informal caregiver burden and healthcare professional’s self-efficacy to provide palliative care. The implementation process will be investigated by a comprehensive mixed-methods evaluation assessing the following implementation constructs: context, reach, dose delivered, dose received, fidelity, implementation level, recruitment, maintenance and acceptability. Furthermore, determinants to implementation will be investigated using the Consolidated Framework for Implementation Research.

**Discussion:**

The COMPASSION study will broaden knowledge on the effectiveness and process of palliative care integration into COPD-care. Furthermore, it will improve our understanding of which strategies may optimize the implementation of integrated palliative care.

**Trial registration:**

Netherlands Trial Register (NTR): NL7644. Registration date: April 7, 2019.

## Background

Chronic Obstructive Pulmonary Disease (COPD) is a common illness characterized by persistent respiratory symptoms and airflow limitation [1]. When the disease progresses, many patients experience recurrent acute exacerbations, often requiring hospital admissions with a mortality rate of up to 23% within 1 year after admission [2]. COPD is the third leading cause of death in the world [3]. Moreover, in advanced stages of the disease, patients suffer from multiple symptoms, which are frequently undertreated [4]. Accordingly, their health-related quality of life is comparable to, or even worse than that of patients with advanced lung cancer [5]. Thus, patients with COPD have at least a similar need for palliative care.

In line with the WHO definition, palliative care should not be restricted to reactive care in the terminal phase of the disease [6]. Instead, it should be provided proactively and earlier in the course of the disease, complementing disease-modifying care. Its goal is to enhance quality of life through assessment and treatment of physical, psychological, social and spiritual problems. Additionally, it is advocated that palliative care should take into account the maintenance of the patient’s autonomy, access to information and treatment options [7], which requires ongoing communication including advance care planning and care coordination. In accordance with current recommendations, palliative care provision should principally be provided by generalist care professionals, i.e. general practitioners and respiratory care specialists in the case of COPD-care, whereas patients can be referred to specialist palliative care if needed [7, 8]. Consequently, palliative care should be an integrated part of regular COPD-care, in which professionals collaborate in multidisciplinary teams to optimize continuity of care.

However, it is not yet clear how palliative care can be successfully integrated into COPD-care. At the moment, discussions on prognosis, goals of care and advance care planning rarely occur or only at a very late stage of the disease [9]. Neither are patients with COPD regularly referred to specialist palliative care [10]. As a consequence, patients with COPD are less likely to die at their preferred place of death, and symptoms remain undertreated [4, 11]. Previous research revealed three major barriers to palliative care provision in COPD [9, 12]. First, the unpredictable disease trajectory of COPD makes it difficult to determine when to start palliative care and discuss advance care planning. The second barrier is the lack of palliative care communication skills of professionals. The third barrier is related to a lack of care continuity and collaboration between healthcare professionals [12, 13].

In the COMPASSION (a central element in the provision of COPD-care [13] and acronym for COPD Palliative and Supportive care Implementation) study, we attempt to overcome these barriers towards the implementation of palliative care. In collaboration with patient and professional organizations, we developed an integrated palliative COPD care intervention that integrates existing scientific and practical knowledge. Moreover, to facilitate uptake of the intervention among healthcare professionals, a multifaceted implementation strategy was developed comprising a training, an online toolbox and support with planning and monitoring of implementation.

In this article, we will describe the aim, design and procedures of the COMPASSION study. Both the implementation process and clinical effectiveness of the integrated palliative care approach will be assessed. The COMPASSION study aims to:

1. investigate the effect of the implementation of integrated palliative care on patient, informal caregiver and healthcare professional outcomes;
2. investigate the effect of the multifaceted implementation strategy on implementation outcomes and explore what barriers hamper the implementation of integrated palliative care in routine COPD-care;
3. explore the relationship between implementation level and patient outcomes.

## Methods

### Design

We follow an effectiveness-implementation hybrid design type 2, as proposed by Curran et al. [14], which allows us to simultaneously test the implementation strategy and impact of the integrated palliative care intervention on health outcomes. To study effectiveness, a cluster randomized controlled trial will be performed in eight hospital regions in the Netherlands. Furthermore, the implementation process will be evaluated using mixed methods. Each hospital region will serve as a cluster. Randomization on this cluster level instead of one-to-one randomization was chosen to reduce contamination: it is likely that professionals exposed to the implementation strategy also would treat patients assigned to the control condition differently [15].

### Setting

This study will take place in eight pulmonary care departments of Dutch hospitals that collaborate with affiliated general practitioners, home care organizations and palliative care consultation teams further referred to as ‘hospital regions’. To increase comparability, academic hospitals were excluded.

### Participants

#### Healthcare professionals

Each participating hospital region forms an intervention group consisting of at least one of the following professions: pulmonologist, respiratory nurse of pulmonology care department, palliative care consultant in the hospital, general practitioner specialized in asthma and COPD, general practitioner specialized in palliative care, consultants from the regional palliative care consultation teams. The following professionals can be involved facultatively if present in that region: respiratory nurse in primary care, pulmonologists in training, practice nurse and other relevant professionals.

#### Patients

Patients diagnosed with COPD and admitted to the hospital for an acute exacerbation will be invited to participate in the study. Patients not able to complete questionnaires in Dutch, patients with severe cognitive decline (e.g. dementia) and patients on the waiting list for lung transplantation will be excluded. After completion of the baseline questionnaire, patients will be screened using the Propal-COPD tool [16]. Patients with a positive Propal-COPD score will be included in the effectiveness study. The Propal-COPD tool consists of seven indicators: Medical Research Council (MRC) dyspnea score of 5, Clinical COPD Questionnaire (CCQ) score > 3, forced expiratory volume in 1 s lower than 30% predicted, presence of specific comorbidities, body-mass index lower than 21 kg/m2 or weight loss (> 10% in the last 6 months or > 5% in last month), previous hospitalization for acute exacerbation in the last 2 years (last 2 years ≥2 admissions or last year ≥1 admission), and a negative answer to the surprise question (“Will you be surprised if your patient would die in the next coming 12 months?”) [16]. For each indicator, specific weight is given, together generating a total score. A score exceeding the previous published cut off value of − 1.362 corresponds with a high probability for death within 1 year, which is considered a proxy for having palliative care needs.

#### Informal caregivers

Informal caregivers of included patients will be invited to participate by asking the patient to indicate who gives him or her the most help and support at home.

### Recruitment of regions

To recruit hospital regions for participation, invitational letters will be sent to the heads of departments of respiratory medicine of all hospitals in the Netherlands. After agreement to take part in the study, eight hospital regions will be selected for randomization to the intervention or control group. Participating regions will be offered a small reimbursement of expenses (maximum €2.5 K per region).

### Randomization

Hospital regions will be randomly allocated by an independent statistician to the intervention or control condition, stratified by the number of COPD exacerbation hospital admissions per year.

### Blinding

To minimize response bias, patients and informal caregivers will not be told whether their hospital is assigned to the intervention or control group. Also, the researcher will be blinded during the analysis process of effect outcomes, using recoded identification numbers of participants. Blinding of professionals for cluster group allocation will not be possible due to the nature of the implementation strategy and intervention. However, professionals of control regions will be blinded for the Propal-COPD score. As a consequence, it remains unknown to them which patients take part in the effectiveness study and receive follow-up questionnaires.

### Project organization

This study is part of a national project coordinated by the Lung Alliance Netherlands, in cooperation with the Leiden University Medical Center and the Radboud University Medical Center, with financial support from The Netherlands Organization for Health Research and Development (ZonMw). A steering group for coordination and three expert groups is set up, representing the relevant patient, family and professional organizations in the Netherlands. In the development phase of the project, every 6 to 8 weeks meetings took place in which the three expert groups gave input on (i) patient-professional communication and compassionate care, (ii) identification of palliative care needs and professional’s expertise, and (iii) implementation and future dissemination, respectively. Additionally, existing practical tools and useful links were selected for an online toolbox supporting professionals.

### Intervention

**Fig. 1:**
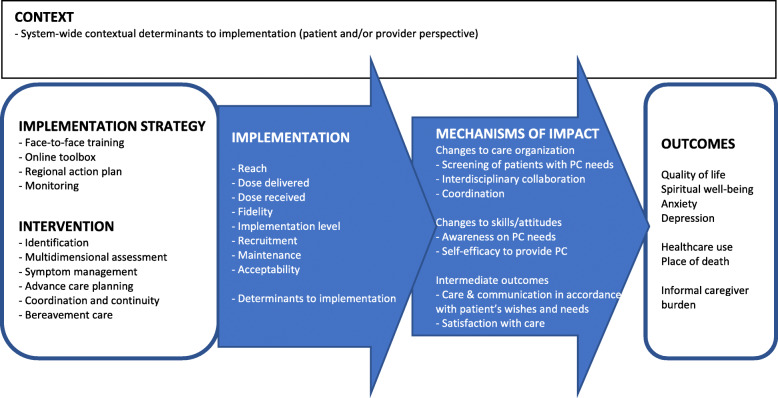
Medical Research Council derived model [26] illustrating causal assumptions of outcomes and mechanisms of impact. Abbreviation PC: palliative care

Table 1 details the components of the integrated palliative care approach, consisting of an integrated palliative care intervention and an multifaceted implementation strategy. Figure 1 shows the causal assumptions of its outcomes and mechanisms of impact, based on the Medical Research Council framework [26]. The integrated palliative care intervention developed follows existing palliative care guidelines [27, 28], the Quality Framework Palliative care of the Netherlands [29], a literature review and input from the expert groups. For the identification of patients that are likely to benefit from palliative care, the previously validated Propal-COPD tool will be used. This tool has been validated in patients admitted to the hospital for acute exacerbation and showed to have a high sensitivity of 90% and specificity of 73% [16]. Moreover, admission for an acute exacerbation of COPD is considered to be one of the key milestones for transition towards a palliative approach [30] and seems to be a feasible moment to start proactive palliative care [31, 32]. After identification, one or more consultations take place either in the outpatient clinic or in the general practice, depending on the patient’s needs, for a multidimensional assessment, symptom management and advance care planning. The treatment plan and agreements made will be documented and shared with other involved professionals and discussed in a multidisciplinary meeting if needed. In case of preference for care at home, the responsibility will be transferred to the patient’s general practitioner. If a patient deceases, the provided end-of-life care will be evaluated with the patient’s informal caregiver and involved healthcare professionals.

**Table 1:** Description of the implementation strategy and integrated palliative care intervention of the Compassion study

**Implementation strategy**		
***Components***	***Content of the component***	***Tools/materials/ underpinning theory***
Formation of regional intervention group	Multidisciplinary regional team	Implementation strategies integrated into multiple settings and directed to multiple professions involved are more effective [17]
Access to online toolbox	Website with information and guidance on the core elements of palliative care in COPD, including tools and links for facultative use	Quality Framework [7] Input from experts
Training session 1 (3 h)	Introductory information on the project and research	n.a.
	Instruction the Propal-COPD tool to identify the palliative phase in patients with COPD	Propal-COPD tool [16]
	Multidimensional assessment (physical, psychological, social, spiritual)	Adapted version of Problems Square [18]
	Communication training on advance care planning in COPD including roleplay with actors	Training in palliative care communication with roleplay supports implementation [19–21]
	Non-pharmacological and pharmacological dyspnea management	Breathing Thinking Functioning model [22]
Training session 2 (3 h)	Discussion current palliative care as organized in region vs. desired palliative care	7-phase model [23]
	Introductory information on implementing care pathway	7-phase model [23]
	Filling in formats A to E (who does what how and when) leading to first draft of regional action plan	Flowchart on patient care process (see Fig. 2)
	Assigning local implementation leaders	7-phase model [23]
Completion of regional action plan	Agreement on who does what how and when	Format regional action plan Action planning stimulates behavior change [24] and assures the suitability of the intervention to the existing structure of the region
Monitoring	Monitoring meetings on site	Audit and provide feedback to monitor, evaluate, and modify provider behavior [25]
	Evaluation meetings with local implementation groups	Share local knowledge on how implementers and clinicians made something work in their setting and then share it with other sites [25]
**Integrated palliative care intervention**		
***Components***	***Content of the component***	
Identification	Calculation of Propal-COPD score	
	Planning first consultation with patient and informal caregiver	
Multidimensional assessment	Assessing palliative care needs on physical, psychological, social and spiritual dimension	
Symptom management	Non-pharmacological and pharmacological treatment for breathlessness and other physical symptoms, smoking cessation, medication review, anxiety and depression	
Advance care planning	Education about the illness trajectory and discussions with patient and informal caregiver on goals and preferences for future medical treatment	
Coordination & continuity	Individual care plan, documentation of advance care directives	
	Information exchange and cooperation with general practitioners and other involved professionals	
	Regular multidisciplinary meetings	
Dying phase & bereavement care	Planning a consultation with informal caregiver to evaluate care in the last phase	
	Planning an evaluation of the provided palliative care with all involved professionals

**Fig. 2:**
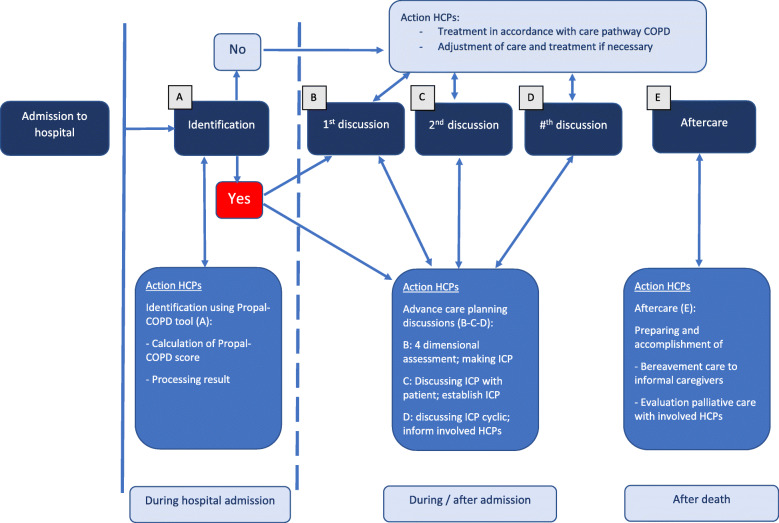
Flowchart on the care process for the individual patient

To facilitate the uptake of the integrated palliative care intervention, an implementation strategy was developed that consists of multiple components [17]. An online toolbox describing the core elements of integrated palliative care and providing easy access to validated, existing tools was established. Also, we developed an interactive training including roleplay for participating healthcare professionals. The training consists of two sessions of 3 h and addresses the core elements of integrated palliative care and its implementation. We collaborated with experienced training actors whose training sessions have been well received in previous research on advance care planning in dementia [33]. Also, the adapted version of the earlier developed Problems Square was used [18]. This tool is a practical translation of the WHO palliative care definition and helps professionals structuring the inventory of actual and possible future problems, and needs and wishes across multiple dimensions. Furthermore, non-pharmacological and pharmacological dyspnea management was discussed. In the second training session, each region collaboratively decides who should be involved, and which steps need to be performed by whom, how, where and when, leading to a regional action plan. In this plan, the different steps of the patient care process are elaborated (see Fig. 2). In order to guide and monitor the implementation and execution of the regional action plans, there will be regularly monitoring meetings with the project leaders of each intervention region. During the study period, every 4 months meetings with participating professionals of the intervention regions will take place in which experiences and recommendations can be exchanged.

Following the recommendations of Mohr et al. [34], we will not evaluate a locked-down version of the intervention, but the implementation of the essential, core elements of the integrated palliative care intervention that will be responsible for the intervention effect. This means that healthcare professionals of all participating settings are allowed to fine-tune the intervention to organizational, professional and patient characteristics. This iterative refinement will result in continuous improvement of the intervention during the study period. Similarly, small adaptations to the online toolbox can be made based on incoming process evaluation data [34].

### Control

Healthcare professionals of the control group will provide care as usual. For the Netherlands, this means that all healthcare professionals have online access to all existing guidelines on palliative care (including dyspnea) and COPD, possibility to consult specialized palliative care teams in primary care as well as in hospitals. After the recruitment of participants has been completed, professionals of the control group will be offered similar training as the intervention group received, and they will get access to the online toolbox.

### Procedures

**Fig. 3:**
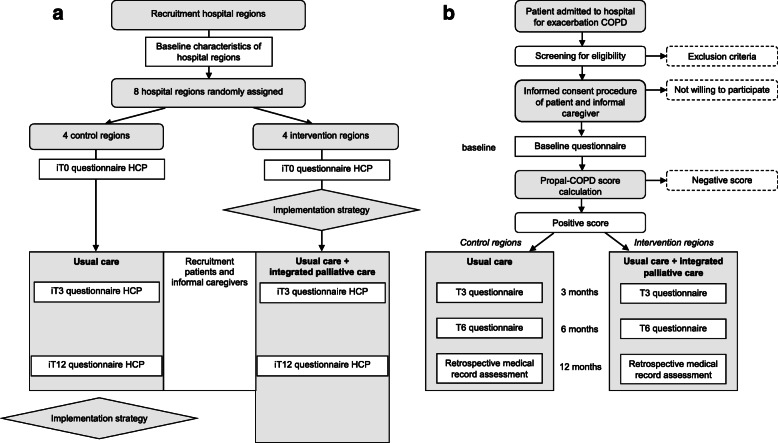
Flowchart of procedures with timelines of (**a**) healthcare professionals and (**b**) patients and informal caregivers. Abbreviations HCP: health care professionals; iT0: professional timeline at baseline (pre-implementation); iT3: 3 months after inclusion of first included patient (initial implementation); iT12: 12 months after training (intervention regions) or inclusion of first patient (control regions) (late implementation); T0: patient timeline at baseline; T3: patient timeline at 3 months; T6: patient timeline at 6 months; R: retrospectively

After randomization and formation of a regional intervention team, participating professionals of all eight hospital regions will be sent the baseline questionnaire by email. This will be repeated 3 and 12 months after the training (intervention regions) or the inclusion of the first patient (control regions). A physician or nurse with knowledge of the study will check all COPD-patients admitted to the pulmonology care department due to an acute exacerbation, and their informal caregiver if applicable, for eligibility. After the informed consent procedure, participating patients and informal caregivers complete the questionnaires required for the Propal-COPD tool and the baseline questionnaires during the hospital stay. A physician or nurse will enter the Propal-COPD tool indicators and baseline characteristics in an online data management system. The system automatically calculates the Propal-COPD score, based on the published algorithm [16]. For a patient of the intervention region, the score will be displayed as “positive” or “negative” and in the control regions it will be displayed as “xxx”. A patient with a positive Propal-COPD score and, if present, his or her participating informal caregiver, will be sent follow-up questionnaires 3 and 6 months after inclusion. Questionnaires will be sent on paper or digitally via email, depending on personal preference. A flowchart of the cluster randomized controlled trial is shown in Fig. 3.

### Effect evaluation

An overview of the data collection process, detailing the timing of data collection and the outcome measures and instruments used, is provided in Table 2.

**Table 2:** Data collection schedule and measurement instruments for patients, informal caregivers and healthcare professionals

	Measurement instrument				
**Patient**		**T0**	**T3**	**T6**	**R**
Baseline characteristics	Questionnaire on demographic characteristics and case report form on disease severity	x			
Quality of life (primary outcome)	Functional Assessment of Chronic Illness Therapy-Palliative care (FACIT-Pal) scale [35]	x	x	x	
Spiritual well-being	Functional Assessment of Chronic Illness Therapy - Spiritual Well-Being (FACIT-Sp-12) scale [36]	x	x	x	
Anxiety and depression	Hospital Anxiety and Depression Scale (HADS) [37]	x	x	x	
Disease-specific health-related quality of life	Clinical COPD Questionnaire (CCQ)	x	x	x	
Satisfaction with care	Single item question on satisfaction with provided care, self-rated on a numeric rating scale (NRS) from 0 to 10	x		x	
Unplanned healthcare use	Medical record assessment on number of ED visits (without admission), hospital admission (number and number of days), ICU admission (number and number of days), in the 12 months pre-enrollment up to 12 months after enrollment				x
Date and place of death, place of care in last week of life if applicable	Medical record assessment (and contact with general practitioner if needed)				x
Dose received	Questionnaire on received core elements, based on three validated questionnaires [38–40]	x		x	
	Medical record assessment on core elements				x
Experiences and acceptability	Semi-structured interviews		x^a^		
**Informal caregiver**		**T0**	**T3**	**T6**	
Baseline characteristics	Questionnaire on demographic characteristics	x			
Caregiver burden	Caregiver Reaction Assessment (CRA) scale [41]	x	x	x	
Satisfaction with care	Single item question on satisfaction with provided care to the patient, self-rated on a NRS from 0 to 10	x		x	
Experiences and acceptability	Semi-structured interviews		x^a^		
**Health care professional**		**iT0**	**iT3**	**iT12**	
Self-efficacy	End-of-life professional caregiver survey (EPCS) [42]	x	x	x	
Role identity	Developed five-item question on role identity based on MIDI questionnaire [43]	x	x	x	
Satisfaction with care	Single item question on satisfaction with provided palliative care to patients with COPD, self-rated on a 5-point Likert scale	x	x	x	
Dose delivered	Self-reported provision of delivered core elements	x	x	x	
Experiences, acceptability and determinants to implementation	Semi-structured interviews			x	

*T0* patient timeline at baseline; *T3* patient timeline at 3 months; *T6* patient timeline at 6 months; *R* retrospectively; *iT0* pre-implementation (professional timeline at baseline); *iT3* initial implementation (3 months after inclusion of first patient); iT12 = late implementation (12 months after training (intervention regions) or inclusion of first patient (control regions))

^a^ Interviews will be held with a purposeful sample of patients and informal caregivers between 3 and 6 months after inclusion

### Primary effect outcome

The primary outcome is quality of life as measured with the Functional Assessment of Chronic Illness Therapy-Palliative care (FACIT-Pal) scale. This scale is a validated 46-item questionnaire comprising of a general part with four subscales on physical well-being, social/family well-being, emotional well-being and functional well-being, respectively, and a specific part regarding palliative care [35]. The total score ranges from 0 to 184; a higher score indicates a better quality of life. Since a Dutch version of the FACIT-Pal questionnaire was not yet available, the items of the palliative care subscale were first translated in collaboration with the FACIT organization, following the FACIT translation/linguistic validation methodology [44]. After a comprehensive forward-backwards translation process, the concept version of the translated FACIT-Pal questionnaire was tested through retrospective and cognitive interviews in ten patients: six with very severe COPD, three with lung cancer and one with both COPD and lung cancer. Recruitment of these patients took place in a pulmonology ward, outpatient clinic, as well as a pulmonary rehabilitation center.

### Secondary effect outcomes

Secondary effect outcomes will be spiritual well-being, disease-specific health-related quality of life, unplanned healthcare use, date and place of death if applicable, informal caregiver burden and professionals’ self-efficacy and role identity with providing palliative care and discussing end-of-life. Satisfaction with care will be assessed at the patient, informal caregiver and professional level. The measurement instruments to be used are detailed in Table 2.

### Baseline measures

Demographic characteristics of patients that will be collected are sex, age, ethnicity (western and non-western), marital status, living situation, an education level (high, medium, low), smoking status, pack years. Also, characteristics of disease severity (long term oxygen use, home non-invasive ventilation use, previous ICU admissions, previous mechanical ventilation and opioid use before hospital admission) will be collected. Demographic characteristics of informal caregivers that will be collected are sex, age, education level (high, medium, low) and current job. Demographic characteristics of professionals that will be collected are function, years of experience, palliative care experience and education.

### Sample size calculation

Our primary outcome measure is quality of life measured with the FACIT-Pal. The clinically relevant difference is suggested to be between 4 to 6% of a measure’s overall score for the Functional Assessment of Cancer Therapy (FACT) scores, including the FACIT-Pal [45]. A previous systematic review on palliative care interventions used the 5% midrange bound, which equals 9 points on the FACIT-Pal [46]. Assuming a standard deviation of 25 [47], at least 121 patients per group are required to obtain a power of 80% (two-sided t-test, alpha = 0.05). To adjust for the clustering at hospital level (a previous study found an ICC = 0.01 [32]) and to allow for an additional loss to follow up of 10% a total of 347 patients are needed (44 patients per region).

### Evaluation of the implementation process

In order to evaluate the implementation process and detect barriers and facilitators, we will perform a comprehensive process evaluation. The following implementation constructs will be evaluated, as recommended by Steckler and Linnan [48]: context, reach, dose delivered, dose received, fidelity, implementation and recruitment. Further, the constructs maintenance and acceptability following recommendations of Proctor et al. will be evaluated. A combination of quantitative and qualitative methods will be used. Various data on the implementation process will be collected: drafts of regional action plans, field notes of training sessions, transcriptions of monitoring meetings, project meetings and interviews with implementation leaders and involved healthcare professionals.

### Implementation outcomes

*Context* refers to the larger physical, social, and political environment that either directly or indirectly affects the intervention. To study contextual characteristics of each intervention region, we will use field notes of training sessions, transcriptions of monitoring meetings, project meetings and interviews with implementation leaders and involved healthcare professionals.

*Reach* concerns the degree to which the intended audience participates in the intervention. Reach of the implementation strategy will be reported as the composition of the intervention teams, the absolute number and the proportion of professionals that participated in the training, using the attendance list of the training sessions. Reach of the intervention will be reported as numbers, proportions and characteristics of patients that received the integrated palliative care intervention.

*Dose delivered* is measured as the extent to which core elements of palliative care have been carried out by participating healthcare professionals, using questionnaires before and after the training. This measure reflects the effect of the implementation strategy on the care practices of each professional.

*Dose received* is measured as the extent to which core elements of palliative care are being received by participating patients, using patient questionnaires and medical chart review on received core elements.

*Fidelity* refers to the extent to which a patient was treated in accordance with the regional action plan. Fidelity checklists for each participating intervention patient will be filled out by the involved healthcare professional.

*Implementation* is a composite score indicating the extent to which the intervention has been implemented by professionals and received by patients. To compare the integrated palliative care provision between the intervention and control regions, the implementation score of delivered and received core elements of integrated palliative care will be calculated using the calculation method following Fleuren et al. [43].

*Recruitment* refers to the way we have recruited hospital regions to become involved in the implementation and evaluation of integrated palliative care in COPD.

*Maintenance* is the extent to which core elements of integrated palliative care is embedded in the routine organizational practices and policies.

*Acceptability* is the perception among healthcare professionals, patients and informal caregivers that the integrated palliative care intervention is agreeable, palatable, or satisfactory. We will perform semi-structured interviews with involved healthcare professionals on their experiences with the intervention and suggestions for improvement. A purposeful sample of patients and informal caregivers will be interviewed about their experiences with and perceptions about the care received and the information discussed with their healthcare professionals. Evaluation questionnaires will inquire on experiences with the training and toolbox and suggestions for improvement among participating professionals. The data will be used to make adaptations to the training and toolbox.

### Barriers and facilitators to implementation

The Consolidated Framework For Implementation Research will be used to explore what determinants to implementation are across different contexts. This framework consists of 37 constructs in five major domains: intervention characteristics, outer setting, inner setting, characteristics of the individuals involved, and the process of implementation. We will use the constructs to develop topic lists for semi-structured interviews with involved professionals.

### Data monitoring and management

All quantitative data will be collected using the online data management system Castor edc. For the management of participants, a secured Access database will be used. In case of errors or essential missing data, the participant or the concerning hospital will be contacted. Of eligible non-consenting patients their year of birth, sex and reasons for non-participation will be collected. The qualitative data gathered via monitoring and project meetings and interviews will all be audio-recorded and transcribed verbatim. The transcripts will be pseudonymized. Also, field notes made during training sessions and contact with professionals of the regions will be written out in digital documents. All study data and meta-data will be stored on a secured place in the Leiden University Medical Center for 15 years.

### Analysis

#### Analysis of effect evaluation

Data cleaning and all analyses will be carried out using statistical software that supports multilevel mixed model analyses, such as IMB SPSS Statistics 25. Baseline characteristics of region and study population will be analyzed using descriptive statistics. Continuous variables will be reported as mean and standard deviation and categorical variables as frequency or percentage. The statistical analyses will be done by an intention-to-treat approach. All analysis will be considered significant if α < 0.05. We will analyze differences between the control and the intervention group for the primary and secondary outcomes on the patient, informal caregiver and professional level using multilevel mixed model analyses that will account for the clustered study design (i.e. patients and professionals nested within a region). We will check for eventual unbalances in baseline characteristics and adjust for these variables if needed.

#### Analysis of process evaluation

All quantitative process data will be analyzed using descriptive statistics. We will examine the association between the implementation score and each effect outcome measure at patient level, using linear regression analysis. For this analysis, the dependent variable is the implementation score. Independent variables are patient outcomes at measurement point T6 or T12. Transcriptions of the semi-structured interviews, monitoring sessions and project meetings will be analyzed using inductively and deductively content analysis techniques supported by the qualitative analysis software Atlas.ti. Subsequently, triangulation of quantitative and qualitative results will take place.

### Ethical considerations

The study has been approved by the Medical Ethics Committee (CMO) of the Radboud University Medical Centre (number 2018–4833). Since palliative care is additional to disease-oriented care, we do not expect any risks of participation for patients. The integrated palliative care intervention is patient-centred (adapted to the needs of every individual patient) and based on existing guidelines and thus could be considered as regular care. However, participation in this study will require filling out questionnaires which can be burdensome. To minimize the burden, we therefore limited the length and frequency of used questionnaires. Moreover, when selecting and writing questionnaires and information letters, we took into account that most patients are older and have a low education level.

## Discussion

This study protocol details the implementation and evaluation of an integrated palliative care approach into regular COPD-care in the Netherlands. The outcomes of this large-scale study will add to the evidence base on how to effectively implement palliative care for patients with COPD, a study population which long has been underrepresented in palliative care research [46].

The integrated palliative care approach was co-created with a wide variety of stakeholders to incorporate scientific and practical knowledge. Also, we built upon previous experiences of national care pathway development for patients admitted to the hospital for an acute exacerbation COPD [49]. Moreover, since coordination between different professionals and transmural collaboration are vital requirements to provide good palliative care, our approach integrates COPD-care and palliative care and includes primary and secondary care professionals.

The effectiveness of integrating palliative care into regular COPD-care can only be tested when it is implemented in a real-world setting. An ‘implementation momentum’ had been created by the publication of the Quality Framework for palliative care in 2017, prescribing the organization and provision of palliative care in the Netherlands, independent of the type of disease [7]. Therefore, a hybrid study design which blends clinical effectiveness and implementation research, as proposed by Curran et al. [14], was chosen. A hybrid design might enable a more rapid translation of our research findings into routine practices, as it will provide information on both the prerequisites of integrating palliative care in routine COPD care and its clinical effectiveness. We are planning to disseminate the study findings and promote the scale-up of the approach if proven effective.

This study will have some methodological challenges. In hybrid effectiveness-implementation studies, implementation science terminology and methods need to be aligned to those of traditional, clinical effectiveness research. Regarding the design, a complex balance needs to be found between internal validity and factors that promote implementation. In our study, to facilitate uptake, the intervention will be tailored to regional needs. Although this resembles real practice and enables generalizability, the heterogeneity of provided intervention elements to patients may limit internal validity.

Furthermore, all hospital regions – including those randomized to the control group – wanting to participate in this study had a particular interest in this topic and were eager to change their care practice. Hence, this might lead to smaller differences between the provided care in the intervention and control group, and may decrease chances of detecting actual intervention effect. We attempt to deal with this by measuring and comparing the implementation score of delivered and received core elements of integrated palliative care between intervention and control groups.

Finally, it is uncertain what proportion of patients will score positively on the Propal-COPD tool and thus will be eligible for inclusion, as we made an assumption based on the development study [16]. Although the Propal-COPD tool is considered to be the best choice because it showed high sensitivity, its robustness and its feasibility in clinical practice need further testing. Therefore, we are planning to perform external validation with the data derived from this study.

## Data Availability

Not applicable.

## Abbreviations

COPD: Chronic obstructive pulmonary disease
FACIT-Pal: Functional Assessment Chronic Illness Therapy; Functional Assessment of Chronic Illness Therapy-Palliative care
RCT: Randomized controlled trial
